# When Heat Meets Pollutants: Integrating Degree-Days and Chemical Activity Concepts for the Assessment of Temperature-Driven Toxicity

**DOI:** 10.1021/acs.est.5c10332

**Published:** 2026-02-20

**Authors:** Elena Gorokhova, Gastón Alurralde, Sophie Steigerwald, Sebastian Abel, Yves Saladin, Anna Sobek, Ann-Kristin Eriksson-Wiklund, Andrius Garbaras

**Affiliations:** † Department of Environmental Science, 7675 Stockholm University, Sv. Arrhenius v. 8, 106 91 Stockholm, Sweden; ‡ Baltic Marine Environment Protection Commission, HELCOM, 00160 Helsinki, Finland; § Mass Spectrometry Laboratory, Centre for Physical Science and Technology, Sauletekio al. 3, Vilnius 10257, Lithuania

**Keywords:** chemical activity, Daphnia magna, degree-days, hydrophobic organic contaminants (HOCs), stable isotope analysis (SIA), temperature-dependent toxicity

## Abstract

Aquatic organisms face simultaneous stress from rising temperatures and chemical contaminants, yet ecotoxicological assays rarely account for temperature-driven physiological aging. We introduce a framework combining degree-days (D°) and chemical activity to disentangle physiological and chemical drivers of toxicity. This approach was tested using *Daphnia magna* exposed to polycyclic aromatic hydrocarbons (PAHs). Two exposure designs were compared: (1) fixed duration (72 h) at 20 and 25 °C, and (2) D°-standardized exposure corresponding to 60 D° (72 h at 20 °C; 58 h at 25 °C). Chemical activity, a thermodynamic measure of bioavailable dose, was used as the exposure metric, with the median lethal activity (La50) as the primary endpoint. To provide a more nuanced view of the metabolism, sublethal responses (δ^13^C, C/N ratio, protein content) were also evaluated. In fixed-time exposures, La50 values were lower at 25 °C, indicating greater apparent toxicity at elevated temperature. This difference disappeared under D° normalization, showing that increased mortality reflected faster physiological aging rather than altered PAH behavior. Metabolic indicators supported this interpretation, revealing PAH-driven, temperature-independent energy depletion. By aligning exposure with cumulative thermal experience and quantifying dose as chemical activity, this framework enables temperature-normalized toxicity assessment and supports climate-aware ecological risk evaluation for HOCs.

## Graphical abstract

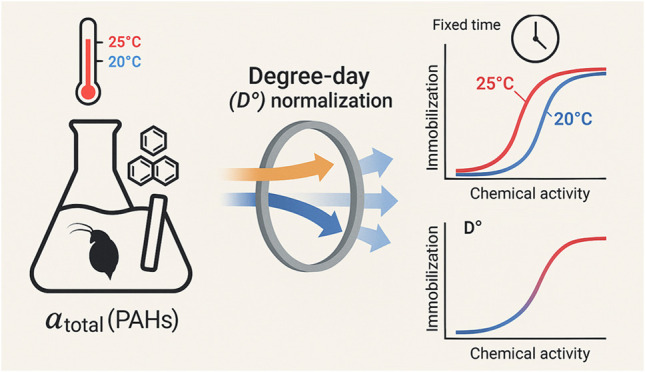

## Introduction

In aquatic environments, organisms are exposed to multiple stressors, including chemical contaminants and rising temperatures.^1,2^ Elevated water temperatures can accelerate contaminant uptake, alter metabolism, and shorten time-to-toxicity,^3^ while bidirectional effects also occur due to differential temperature influences on metabolic uptake and elimination processes.^4^ Temperature is a fundamental factor that not only regulates key biological processes, such as development and growth, but also governs contaminant dynamics by altering the physical-chemical properties of contaminants and cell membranes and influencing pathways involved in their uptake, metabolism, and elimination. Temperature can also influence chemical degradation pathways, potentially creating toxicological profiles for parent compounds and their breakdown products.^5^ Consequently, incorporating temperature variability into toxicity testing is critical for identifying interactive behaviors and ecological risks.

Mechanistic toxicokinetic and toxicodynamic (TK-TD) modeling, including physiologically based toxicokinetic (PBTK) and Dynamic Energy Budget (DEB) models, provides dynamic tools for predicting how exposure and temperature jointly shape toxicity thresholds, body burdens, and organismal responses.^6–9^ These models can, in principle, capture temperature-dependent processes by incorporating changes in metabolic rates and reaction kinetics. However, in experimental ecotoxicology, temperature effects are often tested using fixed-duration exposures, which do not reflect the acceleration of biological aging and metabolism at higher temperatures. As a result, the empirical data generated under such designs may not adequately represent the dynamic processes that TK-TD models aim to describe, thereby limiting their parametrization and validation. This limitation also hampers the ability to distinguish temperature-driven physiological effects from contaminant-specific chemical effects, as both are confounded when exposure duration is expressed in chronological rather than physiological time.

The degree-day (D°) approach provides a means to resolve this mismatch by accounting for temperature-driven biological processes, such as metabolic rates and aging, and enabling exposure comparisons based on physiological rather than fixed time.^10^ Quantifying cumulative thermal exposure helps to separate metabolic changes caused by temperature from the intrinsic potency and reactivity of the toxicants. Using this approach, Cedergreen and co-workers found enhanced metal toxicity at low temperatures due to mechanisms beyond metabolic modulation,^11^ whereas Honkanen and co-workers showed that higher temperatures accelerated the rate at which retene affected trout embryos, without altering the effect concentration.^12^ Such distinctions improve the alignment of empirical data with mechanistic models and enhance the interpretation of temperature effects in both modeling and risk assessment contexts.

Hydrophobic organic contaminants (HOCs), such as polycyclic aromatic hydrocarbons (PAHs), occur in mixtures that are expected to act additively via nonpolar narcosis.^13,14^ Temperature modulates the freely dissolved (bioavailable) fraction and partitioning of PAHs. As the temperature increases, PAH solubility in water tends to rise, and partition coefficients decline, reducing PAH potential to accumulate in biotic lipids. However, warmer temperatures may still facilitate faster chemical diffusion and membrane permeability, which could accelerate the onset of narcotic effects, even if overall internal concentrations do not necessarily increase. Consequently, temperature shifts can modulate both the exposure kinetics and toxicodynamic responses to HOCs.

These effects can be quantified using chemical activity, a dimensionless metric of a contaminant thermodynamic potential to partition into biological tissues, which integrates the individual contributions of multiple HOCs into a single standardized measure of bioavailability.^15^ When the water solubility of HOCs decreases at lower temperatures, a chemical activity in water at a given concentration correspondingly increases.^16^ By reflecting temperature-dependent shifts in solubility and partitioning, chemical activity, as a dose metric, allows an assessment of how the thermal regime affects organismal responses and mixture toxicity. This unified approach provides a means for testing temperature–chemical interactions in ecotoxicity assays with HOCs.

While measures such as chemical activity and lethality quantify exposure and its outcomes, they offer limited insight into the physiological mechanisms underlying toxicity. To better understand how temperature and contaminants affect organismal conditions, additional indicators of metabolic stress are needed. Changes in elemental, isotopic, and biochemical composition of the test animals can reveal shifts in energy use and physiological deterioration during exposure. Among these, the stable carbon isotope signature (δ^13^C), the carbon-to-nitrogen (C/N) ratio, and individual protein content are particularly useful for assessing how key biochemical reserves are mobilized under thermal and toxicant stress.^17–21^

Isotopic fractionation of carbon provides a measure of metabolic turnover. In the absence of food, daphnids rely on endogenous reserves, preferentially catabolizing lighter carbon isotopes (^12^C),^22,23^ leading to δ^13^C enrichment.^24^ Elevated temperature further accelerates this process by increasing respiration and energy demand^25,26^ and preferential catabolism of lighter carbon isotopes.^18,27^ Under narcotic stress induced by HOCs, however, metabolism may slow and δ^13^C values decline, reflecting reduced carbon turnover.^20^ Thus, shifts in δ^13^C integrate both energetic- and toxicant-induced changes in metabolism.

C/N ratios and individual protein content provide complementary perspectives on metabolic allocation.^17^ A declining C/N ratio indicates preferential use of carbon-rich macromolecules, such as lipids,^19^ while reduced protein content signals progressive structural catabolism and energy limitation.^20^ Under moderate thermal stress, the C/N ratio may decline without major protein loss, reflecting increased energy demand, whereas contaminant exposure typically accelerates protein depletion due to impaired metabolic efficiency and detoxification costs. Together, the direction and magnitude of changes in biochemical and isotopic indicators help differentiate temperature-driven physiological responses from contaminant-induced metabolic damage.

Building on these insights, we designed an experiment to disentangle the roles of temperature, physiological aging, and chemical exposure in driving toxicity. Two complementary concepts were integrated: (1) degree-day approach, which aligns exposure durations with cumulative thermal experience and physiological age, and (2) chemical activity, which standardizes exposure in thermodynamic terms and accounts for temperature-dependent changes in contaminant bioavailability. This framework was applied to assess the toxicity of a HOC model mixture of polycyclic aromatic hydrocarbons (PAHs) in *Daphnia magna*. Two exposure regimes were compared: (i) fixed-duration exposures (72 h) at 20 and 25 °C, and (ii) D°-normalized exposures providing an equivalent thermal load of 60 D° (i.e., 72 h at 20 °C vs 58 h at 25 °C). In addition to mortality, we included a set of metabolic indicators (stable carbon isotope signature (δ^13^C), C/N ratio, and individual protein content), to gain mechanistic insight into sublethal effects by tracking shifts in metabolic turnover, macromolecular allocation, and energetic depletion under thermal and toxicant stress.

We hypothesized that (H1) when exposure is expressed in degree-days and dose in terms of chemical activity, toxicity outcomes (median lethal activity; La50) and metabolic responses would not differ between temperatures, indicating that apparent temperature effects arise from accelerated physiological aging rather than changes in contaminant behavior. Conversely, (H2) under fixed-duration exposure, higher toxicity, and more pronounced metabolic stress were expected at 25 °C due to faster depletion of internal energy reserves in unfed animals. If La50 values or metabolic responses differ between temperatures even under D°-normalized conditions, this would signal a true temperature–contaminant interaction, such as altered uptake, biotransformation, or toxicodynamics, demonstrating the added value of combining chemical activity and degree-day metrics for mechanistic toxicological assessment.

## Methods

### Test Organism

We used freshwater cladoceran *D. magna* (environmental pollution test clone 5, Federal Environment Agency, Berlin, Germany), a standard model organism in ecotoxicology. The culture was maintained in M7 medium using standard protocols^28,29^ and fed a 6.5:1 mixture of green algae *Raphidocelis subcapitata* and *Desmodesmus subspicatus* (5–6 × 10^4^ cells/mL) three times per week. Cultures were kept in 2 L glass vessels (20–25 individuals per vessel), 20 ± 1 °C, a 16h:8h light-dark cycle, and weekly medium change. Under these conditions, neonates are released with a body length of 0.9 ± 0.02 mm (mean ± SD; *n* = 45), body dry mass of 4.7 μg/ind. (*n* = 10), and individual protein content of 1.1 ± 0.08 μg/ind. (*n* = 15).

### Chemicals

We used a mixture of four PAHs: acenaphthene (ACE), fluorene (FLU), phenanthrene (PHE), and fluoranthene (FluO), as model hydrophobic organic contaminants (HOCs) to test the hypothesized effects in *D. magna*. These PAHs were selected for their representative hydrophobicity (log Kow: 3.9–5.2) and water solubility (0.1–4 mg L^–1^) ranges for environmentally relevant HOCs in aquatic systems. Stock solutions were prepared by dissolving the PAH crystals (Sigma-Aldrich, St. Louis, MO) in methanol as described in the Supporting Information (SI; Text S1).

### Dose Metrics

Chemical activity provides a standardized representation of the freely dissolved fraction of HOCs, directly linking exposure levels to bioavailability and toxicity potential.^15,30^ It is a dimensionless metric that represents the thermodynamic potential of a chemical to partition and interact within a given matrix, quantifying the biologically active fraction of a contaminant (Text S2, Supporting Information). This integration allows for multiple HOCs to be assessed collectively regardless of differences in their individual concentrations, solubilities, or partitioning behaviors. To account for the temperature effect on PAH solubilities in our system, we estimated solubility values for our congeners at experimental temperatures using the approach developed by Wauchope and Getzen^31^ (Text S2, Table S2; Supporting Information).

Applying chemical activity as a dose metric is based on several key assumptions.^30^ It assumes that the toxicities of individual HOCs are additive for baseline toxicity (narcosis), allowing their combined chemical activities to represent the cumulative toxic potential. Further, the approach relies on equilibrium partitioning between the exposure medium (e.g., water) and biological systems (e.g., cell membranes), ensuring that the chemical activity dosed in the water phase accurately reflects the fraction accumulated in the organism. Finally, baseline toxicity is assumed to be the dominant mode of action in the study system rather than specific or reactive mechanisms.

### Experimental Setup

#### Treatments

Two experimental approaches were applied to assess the combined effects of temperature and chemical exposure: (1) a fixed time design, where daphnids were exposed to contaminants for 72 h at either 20 or 25 °C, and (2) a degree-day (D°) approach, which standardizes exposure based on equivalent physiological age (Table 1). A degree-day is the product of temperature above a chosen baseline and the duration of exposure.^10^ In the D° treatment groups, daphnids were exposed to PAHs for 72 h at 20 °C or 58 h at 25 °C, ensuring both groups received equivalent cumulative thermal exposure of 60 D°. This dual-design framework enabled direct comparisons between fixed-duration exposure and temperature-normalized exposure (Table 1).

**Table 1:**
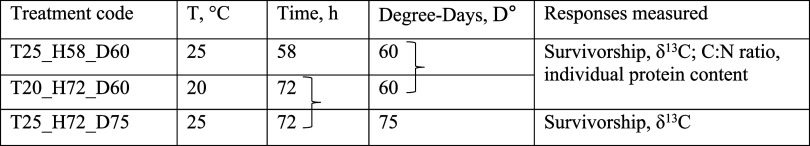
Summary of Experimental Treatments with Corresponding Temperature (T, °C), Exposure Duration (Time, h), and Calculated Degree-Days (D°)^a^

a Treatment codes are formatted to highlight temperature (T), exposure time in hours (H), and degree-days (D). The brackets indicate treatments that were contrasted.

#### Passive Dosing System

A stock solution was used to create the loading solutions for the passive donors, which were used to maintain stable exposure of the PAH mixture (silicone rods; AlteSil Silicone Cord, Altec Extrusions Limited) at five levels of the total chemical activities (nominal values: 0.009, 0.035, 0.045, 0.065, and 0.1). Controls (zero activity, silicone only) were also included in all trials.

All exposure levels were confirmed by measuring PAH concentrations in the pooled exposure media from all vials within each nominal activity level and temperature condition. These measurements provided the average actual concentrations for each combination, from which corresponding chemical activities were calculated (see Table 2; Texts S1 and S2, and Table S1 for details on passive dosing and exposure confirmation). Internal PAH concentrations in daphnids were not measured because the passive dosing system maintained stable chemical activity, ensuring equilibrium between the exposure medium and small (≤5 mg/ind.) *Daphnia*.^32^

**Table 2:** Summary of ACE, FLU, PHE, and FluO Concentrations (mg/L, range) and Corresponding Total Chemical Activities Calculated from the Concentrations as Described in Text S1, Supporting Information, across Different Treatments and Runs^a^

treatment	run	total chemical activity	ACE	FLU	PHE	FluO
T20_H72_D60	1 and 2	0.0075–0.1101	0.023–0.402	0.021–0.399	0.010–0.180	0.0019–0.038
T25_H72_D75	3	0.0037–0.0875	0.017–0.283	0.025–0.322	0.011–0.165	0.008–0.034
T25_H58_D60	4 and 5	0.0083–0.1100	0.030–0.427	0.031–0.378	0.014–0.206	0.0029–0.041

a *Run* refers to an experimental repetition conducted under the specified conditions of temperature, exposure time, and degradation duration, i.e., an independent trial within the same treatment. See Table 1 for temperature and time settings for each treatment and explanations for the treatment codes. In all controls, the measured concentrations were below the detection limit, and activity values were therefore set to zero. Consequently, they are not included in the table.

#### Exposure

Rearing experimental lines and exposure experiments were conducted in climate-controlled chambers. Daphnids were reared at either 20 or 25 °C for three generations using the same medium and food as in the common culture to establish temperature-adapted lines. Neonates (<24 h old) from 2-week-old mothers were used in the exposure experiments, which employed passive dosing with the PAH mixture. Five neonates per vial (20 mL of M7 media) were exposed to six nominal chemical activity levels, including controls, with four to eight replicates per temperature/activity level. The number of replicates varied to maximize the likelihood of obtaining enough survivors for metabolic indicator analysis, particularly at high exposure levels. No food was provided during the exposure, and the neonates were not fed before the experiment, as the early onset of feeding was found to affect animal metabolism and response to PAHs in acute tests.^21^

At the end of the experiment, immobilization was assessed according to OECD guidelines (OECD, 2004) and defined as the inability to move within 15 s of gentle agitation, excluding antennal movement. In line with these guidelines, mortality was calculated by pooling four to eight replicates within each treatment level and expressed as the percentage of immobilized individuals relative to the total number of exposed individuals per temperature/activity level (20–40 individuals). Control mortality remained below 10% in all treatments; the observed mortalities in the exposed groups were adjusted for control mortality using Abbott’s correction.^33^

All surviving daphnids were transferred to screw-cap cryovials by replicate, snap-frozen, and stored at −80 °C. For each temperature/activity combination, 2–5 individuals were first collected for protein analysis, while the remaining 20–35 individuals (127 μg per sample) were pooled and frozen for stable isotope and elemental analysis (total C and, if possible, N). Due to the limited number of survivors, no biological replicates were possible; however, all analyses were run in technical duplicates. When the number of survivors was too low to allow both measurements, stable isotope analysis was prioritized.

#### δ^13^C Signatures

Frozen daphnids from each temperature/activity combination were transferred into preweighed tin capsules, dried at 60 °C, and shipped to the Centre for Physical Science and Technology, Vilnius, Lithuania, for the stable isotope analysis. The analysis was conducted using a Flash EA 1112 Series Elemental Analyzer, which was connected via a Conflo III instrument to a DeltaV Advantage Isotope Ratio Mass Spectrometer (all Thermo Fisher Scientific, Bremen, Germany). The stable isotope ratio of ^12^C/^13^C, denoted by δ^13^C, is defined as the deviation in ‰ from an international reference standard (Vienna Pee Dee Belemnite). An internal reference (cod muscle tissue prepared in the same way as the test samples) was analyzed every 20 samples; the analytical precision for δ^13^C was 0.1‰.

#### C/N Ratio

In concert with stable isotope analysis, the total carbon (C) and nitrogen (N) were measured, and the C/N ratio was calculated and used as a proxy for the relative lipid and protein content.^17–19^ Additionally, since the daphnids had a C/N ratio greater than 3.5, their δ^13^C values were corrected to minimize bias resulting from ^13^C depletion in lipids.^34^

#### Individual Protein Content

Frozen daphnids were homogenized in 130 μL of Potassium-Phosphate-Buffer (PPB) using an MP FastPrep-24 (Nordic Biolabs), 20 s × 3, at 4.5 M/s after adding approximately 40 mg of glass beads (212–300 μm, Sigma-Aldrich). The samples were then centrifuged (3300 rcf) for 5 min at 4 °C, and the supernatant was transferred to clean tubes and stored at −80 °C until further analysis. Protein concentrations were measured using a modified Lowry method implemented in the Micro-BCA Protein Assay Kit (Thermo Scientific). After 120 min of incubation at 37 °C, the absorbance in the samples was analyzed using a microplate reader (FLUOstar OPTIMA, BMG LABTECH) at 562 nm. The standard curve was prepared with Bovine Serum Albumin (BSA). The individual protein content was expressed as micrograms of protein per individual. using measured concentrations, extraction volume, and number of individuals per sample.

### Data Analysis

#### PAH Profiles

A Permutational Multivariate Analysis of Variance (PERMANOVA) was used to assess differences in the relative contributions of PAH congeners among treatments, based on their measured concentrations. The analysis was performed using PRIMER+ (v7, PRIMER-e, Auckland, New Zealand), based on a similarity matrix with 998 unique permutations used to obtain *p*-values. A pairwise PERMANOVA was conducted to test post hoc differences between treatment groups and further investigate potential differences.^35^ This analysis was necessary because different PAH congeners (and their metabolites) have varying specific toxicities in addition to the common mechanism of narcosis.^36^ Some of these effects, such as developmental and cardiotoxic mechanisms in invertebrates, including cladocerans, may be observable at our test concentrations,^37^ making it crucial to ensure that their profiles were comparable across treatments. If no significant differences were detected between treatments by PERMANOVA, this would rule out the possibility that specific effects influenced dose–response relationships, ensuring that the observed effects were due to the baseline toxicity rather than variations in specific toxicities.

Additionally, a Permutational Analysis of Multivariate Dispersions (PERMDISP) was performed to assess differences in the variability of PAH profiles (Table S1, Supporting Information) between treatments.^35^ This test evaluates whether any differences detected by PERMANOVA are due to location shifts in multivariate space rather than disparities in dispersion among the groups. A nonsignificant PERMDISP result, combined with a nonsignificant PERMANOVA result, suggests that the lack of treatment effects (i.e., differences across the experimental groups) is not due to differences in data spread.

#### Lethal Activity Values

Toxicity was quantified as La50 values (the activity causing 50% immobilization) for each treatment. Immobilization data were fitted with a sigmoidal dose–response curve with a variable slope (significance threshold *P* < 0.05) using GraphPad Prism version 10.6.1 (GraphPad Software, LLC) to obtain La50 estimates and their 95% confidence intervals (95%-CI).

For formal comparison among treatments, differences in La50 were evaluated using a joint four-parameter logistic model with shared asymptotes and Hill slope and treatment-specific La50 estimates. This model structure accommodates unequal sample sizes (12 observations per treatment for T20_H72_D60 and T25_H58_D60, and six for T25_H72_D75) and ensures that La50 differences are tested under consistent curve geometry (see Text S4 for details).^38^

The overall treatment effect was assessed by a likelihood ratio test comparing a model with a common La50 to one with treatment-specific estimates. Pairwise contrasts of the Log­(La50) ratios were then evaluated using Wald tests to assess differences between the treatments. In addition, the equivalence between T20_H72_D60 and T25_H58_D60 was tested on the La50-ratio scale using the same model. Following the two one-sided tests (TOST) procedure,^39^ treatments were considered equivalent when the 90% confidence interval for the ratio lay entirely within 0.80–1.25, corresponding to an accepted ± 20% variation regarded as biologically negligible in toxicological assays.^40^

#### Metabolic Responses

Individual protein content, C/N ratios, and δ^13^C responses were evaluated to determine whether biochemical deterioration associated with exposure follows similar patterns across temperature conditions. Because protein content showed a unimodal response, its relationship with dose (expressed as chemical activity) and temperature was evaluated using a Generalized Linear Model (GLM) with a log link and a normal error structure. Prior to analysis, protein values were mean-centered to facilitate interpretation of temperature effects independent of variation in mortality.^41^

In contrast, δ^13^C and C/N ratios exhibited nonunimodal responses and were therefore analyzed using Generalized Additive Models (GAMs) to evaluate the effects of dose and temperature. Before modeling, values were normalized by calculating percent deviation from the control. Heteroscedasticity was addressed using a weight vector based on deviations from the median, and influential observations were identified using Cook’s distance. Nonlinear relationships were modeled using splines, assuming a common effect across temperatures:

$$\delta^{13}C\;\left(\mathrm{or}\;C/N\right)∼s\;\left(d\mathrm{ose},K=4\right)+T$$

Model performance was assessed using adjusted *R* ^2^, deviance explained, Akaike Information Criterion (AIC), and generalized cross-validation (GCV). GAMs were performed in R using the *mgcv* package.^42^

## Results

### Consistency of PAH Mixture Composition across Experimental Treatments

Analysis of PAH mixture composition across five independent runs showed no significant differences among treatments (PERMANOVA: Pseudo-F = 0.5061, *P* = 0.606; Table 3, Figure 1). Homogenous dispersion among treatments (PERMDISP: *F* = 0.068, *P* = 0.953) and nonsignificant pairwise comparisons (all *P* values > 0.77) confirmed that variability in measured PAH concentrations was not treatment-specific. The PCoA plot shows tight sample clustering with PCO1 explaining 99.7% of the total variation. The chemical activity vector aligned along PCO1 reflects minor compositional differences unrelated to treatment effects (Figure 1; Table 3).

**Figure 1:**
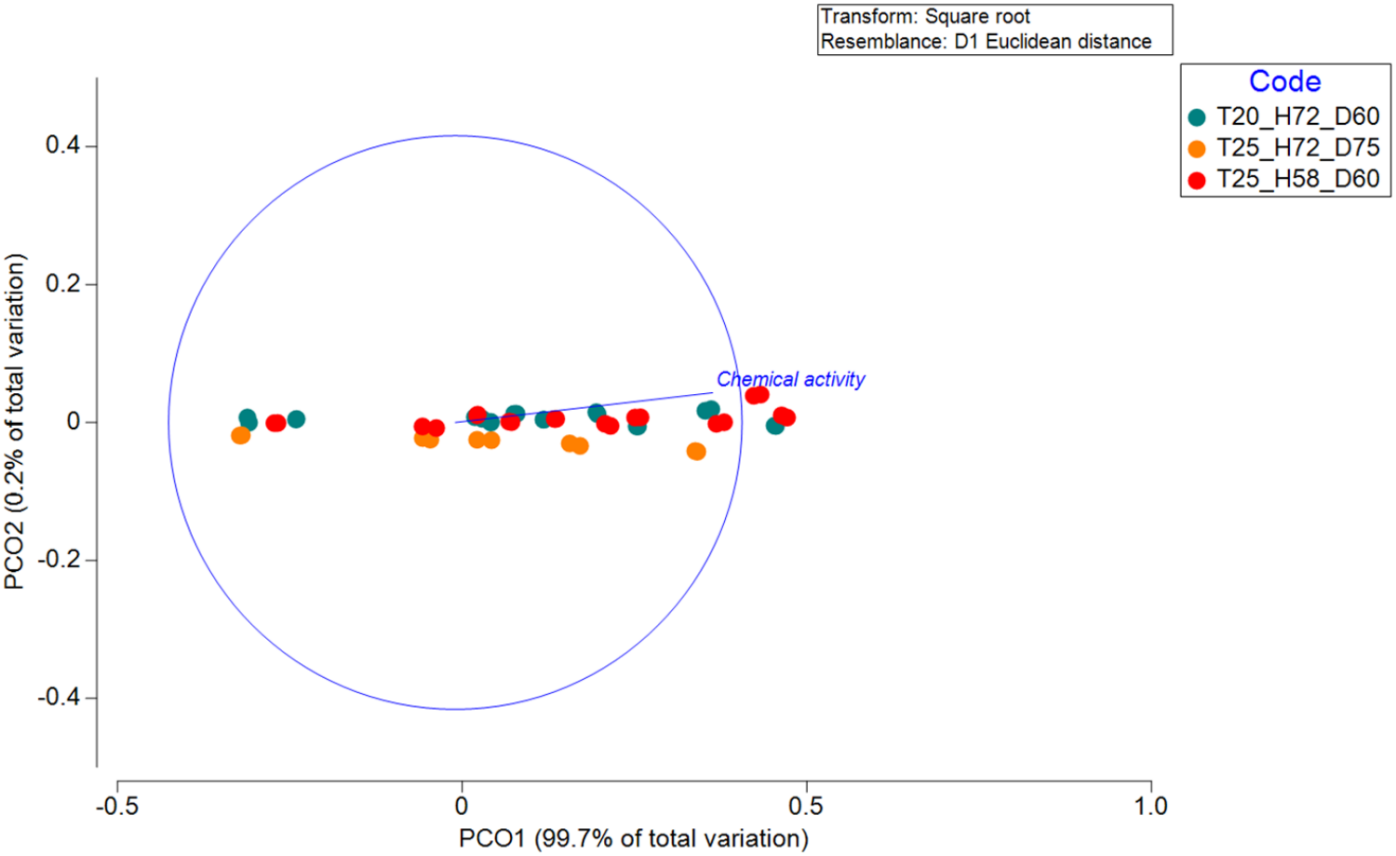
Principal coordinates analysis (PCO) plot based on PAH congener profiles based on their chemical activities across different treatment groups (Code). The analysis was conducted using a square root transformation and a resemblance matrix based on D1 Euclidean distance. The first axis was associated with the total chemical activity and explained 99.7% of the total variation. Colors represent different treatment groups as indicated in the legend. The vector overlay represents the correlation of chemical activity with the ordination space. See Table 3 for the PERMANOVA output.

**Table 3:** Results of PERMANOVA Assessing Differences in PAH Congener Profiles among Treatment Groups

(A) main test results, showing the degrees of freedom (df corresponding to 60 samples; see Table S1), sum of squares (SS), mean squares (MS), pseudo-F statistic, and permutational *p*-value (P(perm)) based on 998 unique permutations						
source	df	SS	MS	pseudo-F	P (perm)	unique perms
treatment	2	0.1105	0.055226	0.5061	0.606	998
residual	57	6.2199	0.10912			
total	59	6.3303				

(B) pairwise comparisons between treatment groups.		
comparison	t	P(perm)
T20_H72_D60 vs T25_H72_D75	0.3320	0.788
T20_H72_D60 vs T25_H58_D60	0.0490	0.972
T25_H72_D75 vs T25_H58_D60	0.3437	0.774

### Temperature Effect on Mortality

The three dose–response curves (Table 4, Figure 2A) all fall within a chemical activity range of 0.01 to 0.1, commonly associated with baseline toxicity for hydrophobic organic chemicals.^30^ The La50 estimates from the individual (Table 4) and joint four-parameter logistic model with shared asymptotes and slope (Table S3, Supporting Information) fits were nearly identical (Figure 2B), and the slight reduction in uncertainty in the joint model, reflect the stabilization of parameter estimates achieved by sharing the asymptotes and slope across treatments. The joint model indicated significant heterogeneity in La50 among treatments (likelihood ratio test, χ^2^ = 34.17, df = 2, *p* < 0.0001). A complementary parametric bootstrap analysis (Table S5) produced overlapping percentile intervals for the same treatment contrasts, confirming that the observed La50 differences and their uncertainties were robust to the model assumptions.

**Figure 2:**
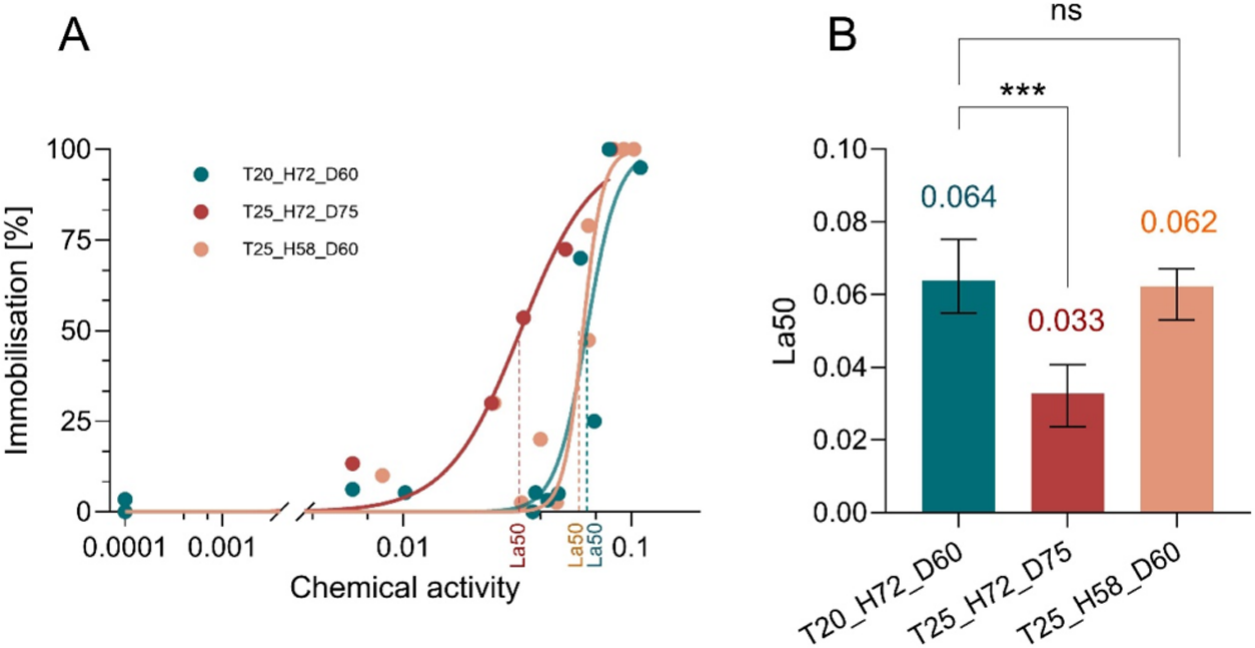
Dose–response relationships and median lethal chemical activity (La50) of *D. magna* under fixed-time and degree-day exposures. (A) Immobilization (%) as a function of chemical activity of the PAH mixture under three temperature/exposure scenarios (see Table 1); curves represent sigmoidal fits of the experimental data with dashed lines marking the La50. The zero-activity control was assigned a small positive pseudovalue (1 × 10^–4^) to allow visualization on a logarithmic *x*-axis; this value has no biological meaning and does not affect the estimation of La50. (B) Estimated La50 values for the three treatments. Bars represent the best-fit La50 (±95% CI) from individual dose–response models (Table 4). See Tables S4 and S5 for the formal comparison of treatments. Significance annotations (***: *p* < 0.001; ns: not significant). The D60 treatments were statistically equivalent within ± 20% according to the TOST criterion (Table S6).

**Table 4:** Summary of Dose-Response Analysis for Immobilization (mortality, %) Data in the Three Treatments (columns) Using a Stimulation Variable Slope Model, as Implemented in GraphPad Prism v. 10.4.0.^a^

model parameters	**T20_H72_D60**	**T25_H72_D75**	**T25_H58_D60**
best-fit values			
hill slope	5.317	2.74	7.418
La50	0.0635	0.033	0.0623
95% CI (profile likelihood)			
hill slope	2.429 to 12.22	1.205 to 5.482	3.111 to 28.40
La50	0.0529 to 0.0767	0.0236 to 0.0407	0.0530 to 0.0707
goodness of fit			
degrees of freedom	10	4	10
*R* ^2^	0.8197	0.9617	0.8821
sum of squares	2928	260.4	2331
Sy.x	17.11	8.068	15.27
normality of residuals			
Shapiro–Wilk (W)	0.890	0.989	0.961
*P* value	0.119	0.988	0.802

a This model extends the standard four-parameter logistic equation applied to each dataset independently; the variable slope (Hill coefficient) accounts for differences in the steepness of the dose-response curves, providing a flexible fit.

The joint model indicated a significant difference in La50 values between T20_H72_D60 and T25_H72_D75 (0.065 vs 0.033; La50 ratio = 1.98, 95%-CI = 1.59–2.45, *P* < 0.001; Table S6), demonstrating that temperature strongly increased immobilization when exposure duration was expressed as chronological time (72 h) rather than normalized by D°. Thus, an elevated temperature exacerbated the apparent toxicity estimate under fixed-time exposure.

In contrast, no significant difference was detected between the temperature regimes under the D°-standardized exposure (T20_H72_D60 vs T25_H58_D60; 0.065 vs 0.061; La50 ratio = 1.07, 95%-CI = 0.90–1.28, *P* = 0.42). According to the TOST, the 90%-CI for the ratio (0.92–1.24) lies entirely within the 0.80–1.25 equivalence bounds, confirming that these treatments are statistically equivalent within ±20%. This equivalence supports that the D° normalization effectively compensates for the higher metabolic expenditure at 25 °C, thereby neutralizing the temperature-related increase in toxicity observed under fixed-time exposure.

### Temperature Effect on Metabolic Responses

δ^13^C values (Figure 3A) decreased nonlinearly with increasing chemical activity, with the steepest decline in the fixed-time/high-temperature group (T25_H72_D75). In the D° treatments, the decline became apparent only above ∼0.05 activity. The C/N ratio (Figure 3B) also decreased at higher activities, and protein content (Figure 3C) declined approximately linearly. In the D° exposures, these responses were driven by dose: chemical activity had a significant effect on all three indicators, whereas temperature did not (Tables 5 and 6). Thus, the dose-dependent metabolic responses were not influenced by temperature when the physiological time was standardized.

**Figure 3:**
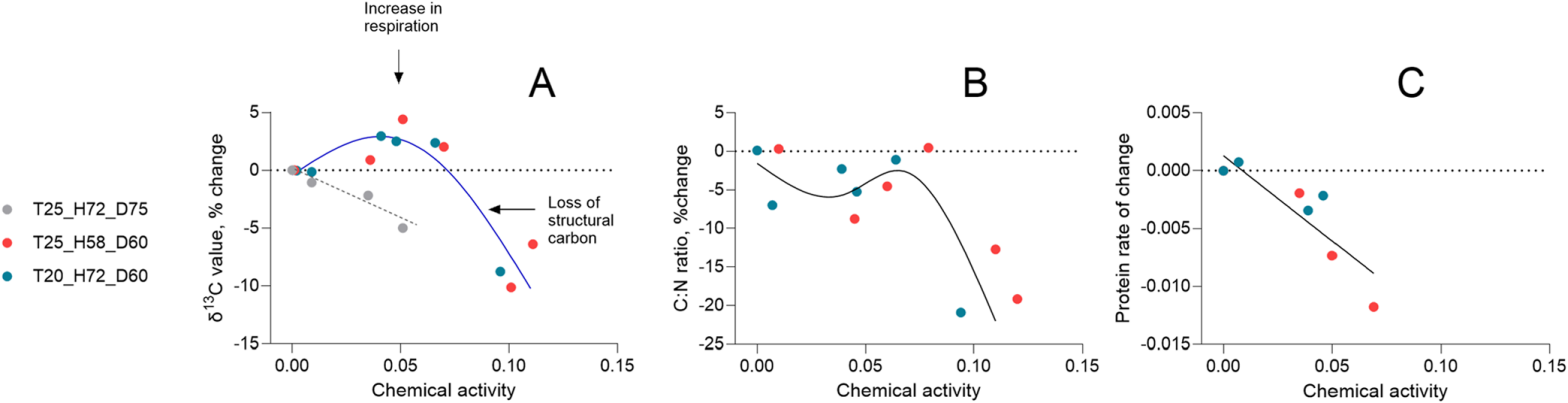
Relationships between chemical activity and metabolic response variables normalized to controls (dotted line) as a function of chemical activity across different treatment groups. (A) Change (%) in δ^13^C values, indicating shifts in carbon metabolism, with potential increases in respiration at lower doses and loss of structural carbon at the highest doses. (B) Change (%) in the C/N ratio, suggesting a decline in lipid storage at lower doses and protein breakdown at the highest doses. (C) Rate of change in individual protein content in relation to the control. Unfortunately, the number of samples for the T25_H72_D75 group was insufficient for the C/N ratio and protein content due to the high mortality rate; therefore, no data is available for the highest doses for these variables. Colors represent different treatment groups as indicated in the legend. Trend lines illustrate nonlinear/linear responses; see Tables 5 and 6 for statistical testing of the treatment effects.

**Table 5:** Summary of GAM Results Testing the Effects of Dose (Expressed as Chemical Activity) and Temperature on the Biochemical Indicators δ^13^C (A) and C/N Ratio (B) for the Degree-Day Exposures^a^

response variable	predictors	df	GAM coef.	standard error	standard score	nonlinear *P* value
(A) δ ^13^C	intercept	1.000	32.774	2.378	13.781	
	dose	4.000	–206.637	36.752	–5.622	<0.0001
	temperature	1.000	1.779	2.666	0.667	
(B) C/N ratio	intercept	1.000	37.144	4.137	8.977	
	dose	4.000	–268.075	63.943	–4.192	0.012
	temperature	1.000	3.168	4.638	0.682	

a The response variables were Box-Cox transformed, and models were fitted with a normal error structure and an identity link. Reported values include GAM coefficients, standard errors, standard scores, and *P* values for nonlinear smooth terms.

**Table 6:** Summary of GLM Results Assessing the Effects of Dose (Expressed as Chemical Activity) and Temperature on Individual Protein Content in the Degree-Day Exposures^a^

predictors	estimate	standard error	wald χ^2^	*p*
intercept	–4.371	0.113	1487.498	< 0.0001
dose	–13.862	4.206	10.859	< 0.0001
temperature	0.092	0.090	1.031	0.309
scale	0.002	0.0005		

a The response variable was Box–Cox transformed, and the model was fitted with a normal error structure and a log link. Reported values include parameter estimates, standard errors, Wald χ^2^ statistics, and associated *P* values

Exploratory plots of the biomarkers against mortality (Figure 4) showed consistent decreases in δ^13^C, C/N ratio, and protein content with increasing mortality, with similar patterns at both temperatures. The steepest changes occurred at activity levels near La_50_, indicating that shifts in metabolic indicators coincided with the range of mortality observed under the D°-normalized conditions.

**Figure 4:**
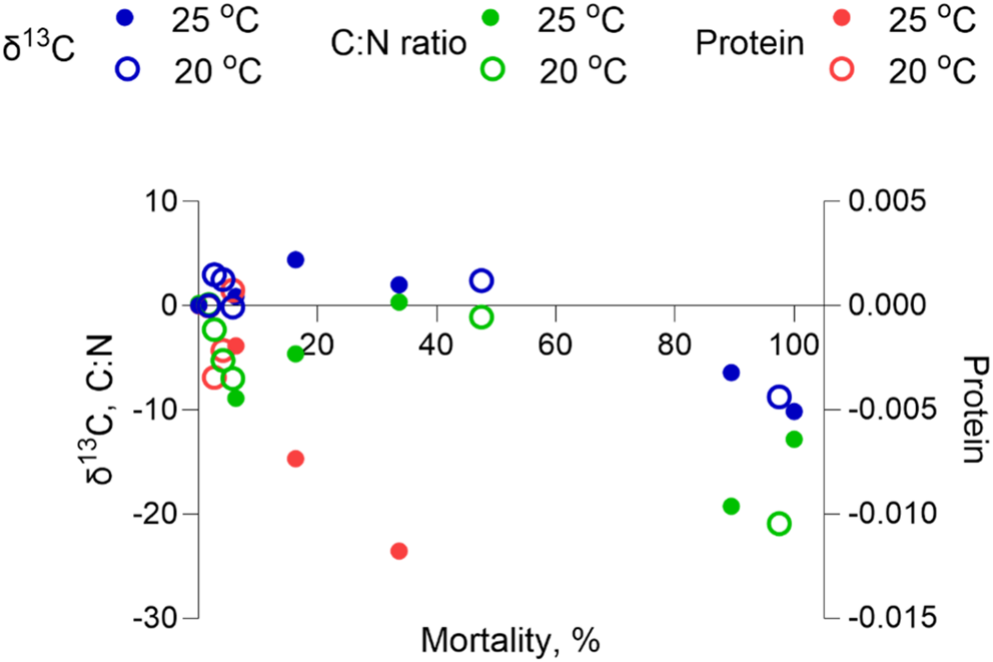
Relationships between biochemical indicators of metabolic activity (Y axes; normalized to controls), δ^13^C values, C/N ratio, and individual protein content (Protein; rate of change) and mortality (X axis; %) at two temperatures (20 and 25 °C) for the degree-day exposures. The declines of all three indicators with increasing mortality suggest physiological deterioration, metabolic shifts, or energy depletion in individuals experiencing higher mortality, reinforcing the link between these indicators and toxic outcomes. The similar rates of decline at both temperatures indicate that the underlying biochemical changes are not temperature-dependent. The left *Y*-axis corresponds to δ^13^C value and C/N ratio, while the right *Y*-axis corresponds to protein.

## Discussion

### Temperature-Dependent Toxicity Effects Disappear under Degree-Day Normalization

The contrast between fixed-time and D°-normalized exposures illustrates how temperature-driven differences in metabolic rate shape the apparent toxicity. Under fixed-time conditions, daphnids exposed at 25 °C exhibited nearly twice the toxicity observed at 20 °C over the 72 h (Figure 2B). In contrast, when exposures were standardized to 60 D°, the temperature-dependent difference in toxicity disappeared. The equivalence analysis further demonstrated that the D°-standardized treatments were not only statistically indistinguishable but also biologically equivalent, confirming that the normalization effectively eliminated the temperature effect rather than merely reduced its detectability.

The convergence of La50 values under D° normalization indicates that the apparent temperature effect was driven by differences in cumulative metabolic aging rather than changes in the mode of action or PAH behavior. This outcome supports our hypothesis and aligns with previous observations in juvenile trout, where temperature influenced the rate but not the mechanism of toxic effects.^12^ In principle, the higher apparent toxicity at 25 °C during fixed-time exposure might result from transiently higher internal PAH accumulation, since uptake kinetics increase with temperature.^7,43^ However, this explanation is unlikely given our equilibrium-based design: the passive dosing system maintained constant chemical activity across treatments, and small *Daphnia* rapidly approach equilibrium with the exposure medium.^32^ The disappearance of temperature effects under D° normalization further indicates that any temperature-related differences in the internal dose were minor. Moreover, at equal chemical activity, higher temperature enhances solubility and reduces partition coefficients,^16^ thereby counteracting rather than amplifying internal accumulation.

All dose–response curves fell within the chemical activity range of baseline toxicity for hydrophobic organic chemicals (0.03–0.10), consistent with narcosis-based toxicity thresholds.^30^ As PAH composition remained consistent across treatments (PERMANOVA; Figure 1), we can rule out differences in mixture profiles or specific toxicities as contributors to the observed La50 variations. Thus, the increased toxicity in fixed-time exposures was primarily due to temperature-driven metabolic changes rather than differential chemical interactions or mechanisms of action.

Extending this approach in future experiments could further clarify the mechanisms underlying the temperature-toxicity interactions. In particular, expanding the design to include a time series of degree-day exposures and treatments with controlled feeding would help disentangle how the energy balance and metabolic rate jointly shape susceptibility and recovery. Such additions would also enable direct quantification of the links between physiological aging, metabolic turnover, and toxic response, providing a dynamic view of toxicity progression. From a statistical perspective, increasing replication within treatments and improving dose resolution around the La50 region would enhance parameter precision and strengthen the robustness of both difference and equivalence testing.

### In Degree-Day Exposures, Mortality Is Driven by Metabolic Exhaustion Rather than Temperature

A key factor in temperature-mediated toxicity is the depletion of energy reserves during exposure with no feeding. Higher temperatures increase the metabolic demand, accelerating the loss of internal resources and potentially compromising detoxification capacity. Initially, lipids serve as the primary catabolic source, followed by proteins as the main energy substrate once lipid stores are exhausted.^18,44^ Elevated metabolic activity also increases oxygen demand, which organisms meet by enhancing ventilation and raising the uptake of waterborne contaminants.^45^ Although lower temperatures offer an energetic advantage by reducing metabolic rates (i.e., a “cold advantage”),^46^ this benefit may be offset by contaminant-induced disruption of oxidative phosphorylation, especially by PAHs and other hydrophobic organic compounds. Moreover, temperature effects on toxicokinetics often lead to higher bioconcentration (and associated toxicity) at lower temperatures, because uptake remains largely^6,47^ passive while depuration is metabolically driven and slows substantially in cold conditions due to reduced enzymatic and excretory activity.^43^ Consequently, metabolism provides a sensitive link between exposure and survival outcomes.

The strong negative associations between mortality and biochemical indicators (δ^13^C, C/N ratio, and protein content) demonstrate that physiological deterioration increases with exposure (Figure 3) and closely tracks the onset of lethal effects (Figure 4). The decline in δ^13^C indicates intensified metabolic exhaustion in the exposed daphnids, which, in the absence of a food supply, preferentially catabolize lighter carbon isotopes under energy-demanding conditions.^22^ The single-phase decline in δ^13^C values observed in the fixed-time/high-temperature group reflects early onset metabolic stress, likely driven by the combined effects of elevated temperature and longer absolute exposure in the absence of food. In contrast, under D°-normalized exposures, δ^13^C signature followed a biphasic trend in relation to both chemical activity and mortality (Figures 3A and 4): a moderate enrichment (∼5%) at lower exposures (< 0.05 chemical activity) likely reflects increased metabolic turnover and possibly detoxification-linked respiration, while a sharp depletion (∼10%) at higher exposures suggests a shift toward catabolism of structural macromolecules (i.e., proteins), consistent with advanced energy depletion.^20^

This interpretation is supported by Valladares and Planas,^48^ who demonstrated that stable isotope signatures in juvenile seahorses aligned more closely with cumulative thermal exposure, expressed as D°, than with chronological age, particularly under feeding constraints. Their finding that δ^13^C and δ^15^N dynamics reflect the balance between assimilation and metabolic demand under thermal stress reinforces our conclusion that δ^13^C shifts in *Daphnia* can serve as integrative indicators of metabolic status across treatments. Therefore, combining D° normalization with isotope-based biomarkers allows for the diagnostics of temperature-adjusted physiological stress in ecotoxicological studies.

Concurrent reductions in C/N ratio (Figure 3B) and protein content (Figure 3C) further highlight a transition to protein catabolism as an energy source, a known response to stress-induced energy limitation and toxic exposure.^49^ As hypothesized (H1), these biochemical responses were not temperature-dependent under D°-normalized exposures (Tables 5 and 6), suggesting that contaminant exposure rather than temperature differences drove physiological stress and detoxification costs. The absence of temperature effects in the δ^13^C, C/N ratio, and protein content under D°-normalized exposures further supports the capacity of this approach to isolate and quantify metabolic versus chemical drivers of toxicity.

### Integration of D° Approach and Chemical Activity for Temperature Effect Assessment

A better understanding of temperature-toxicity dynamics is increasingly emphasized in mechanistic, climate-sensitive ecotoxicology frameworks.^4,8^ Integrating the D° approach with chemical activity metrics offers a unified basis for distinguishing whether temperature-dependent HOC toxicity arises from physiological deterioration linked to energy constraints or from altered contaminant kinetics, such as chemical-membrane interactions that enhance uptake and/or biotransformation.

Traditional classifications of temperature–contaminant interactions as synergistic, additive, or antagonistic^50^ depend on how exposure is defined. In fixed-time tests, higher temperatures accelerate metabolic rates and physiological aging, which can increase toxicity and create patterns that resemble synergistic or additive effects, though temperature itself has no toxic mode of action. As emphasized in multistressor research, diagnoses of synergy or antagonism do not necessarily reflect mechanistic interactions but depend on the chosen null model, and this is particularly relevant when one stressor primarily alters organismal physiology rather than toxicodynamics.^51^ The D° approach addresses this by aligning exposures to cumulative thermal experience, allowing for clearer separation of physiological and contaminant-driven effects. If toxicity at equal D° remains unchanged, then effects are best described as independent rather than truly additive in a toxicological sense. If toxicity increases, this indicates a true synergistic interaction (i.e., temperature-enhanced uptake or bioactivation^52^); if it decreases, antagonistic effects, such as accelerated detoxification, are indicated (Table S7, Supporting Information).

In our study, the absence of temperature-dependent differences under D°-normalized conditions indicates that the interaction between temperature and PAH toxicity is independent, despite the apparent additive interaction seen under fixed-time exposure. Combined with chemical activity, which accounts for temperature-dependent changes in bioavailability and partitioning, the D° framework provides a mechanistically grounded basis for interpreting thermal modulation of toxicity, particularly for nonpolar HOCs acting primarily through narcosis.

These findings were obtained under starvation, where physiological deterioration was dominated by catabolism. In feeding organisms, the temperature also influences ingestion, assimilation, and growth, while HOCs can disrupt these same processes. Consequently, both energy intake and toxic stress act on overlapping metabolic pathways, affecting the overall energy balance. In feeding and growing animals, the combined variability in energy acquisition and contaminant dynamics may increase the complexity and potentially the magnitude of the observed effects. Under such conditions, the D° framework, with chemical activity as its dose metric counterpart, remains valid and can help disentangle physiological and chemical contributions when supported by larger sample sizes, time-resolved measurements, and additional physiological endpoints. Integrating these data with dynamic models that link energy budgets to toxicokinetics and toxicodynamics (e.g., DEBtox or PBTK–TD approaches) will enable evaluation of the net biological effects arising from combined climate and pollution pressures. Such models can already estimate the relative contributions of thermal and chemical stressors by incorporating temperature-dependent rate parameters, but their accuracy remains limited when exposures are defined solely in terms of time. Coupling TK-TD modeling with D° normalization would provide a more mechanistic separation of temperature- and contaminant-driven effects.

In this context, the D°-based experiments provide temperature-aligned empirical data that can constrain and validate dynamic models, simulating contaminant uptake, metabolism, and elimination, under variable environmental (including food availability) conditions.^6–9^ By supplying biologically scaled toxicity endpoints, the D° experiments enable model parametrization that captures realistic thermal physiology. In turn, such mechanistic models can extrapolate experimental outcomes to broader temporal and ecological contexts (Table S8, Supporting Information) advancing temperature-sensitive chemical risk assessments.

#### Implications for Climate-Aware Ecotoxicology

This study demonstrates the value of combining physiological time (degree-days) with thermodynamically consistent dose metrics (chemical activity) to disentangle temperature- and contaminant-driven effects in toxicity testing. By aligning exposure with organismal metabolism, this approach improves the ecological relevance of test designs and mechanistic interpretation, especially when supported by sublethal metabolic indicators. For regulatory ecotoxicology, this framework offers a practical tool for climate-adapted risk assessment. We need more accurate comparisons across temperature regimes that distinguish between chemical and physiological drivers of toxicity and provide transferable metrics for cross-species or system-level assessments. As environmental risk frameworks evolve to account for multiple stressors,^4^ moving beyond fixed-time, concentration-based testing will be essential for realistic evaluations.

## Supplementary Material





## Notes

Raw and processed data for immobilization, mortality, δ^13^C, C/N ratio, and protein content measurements used in dose–response and metabolic analyses (XLSX)
Description of PAH dosing system, analytical verification, and temperature adjustment of chemical activity; degree-day calculation; statistical testing rationale; tables of PAH concentrations, solubilities, and La_50_ estimates; bootstrap and equivalence analyses; and framework for integrating D° normalization and chemical activity into TK–TD models (PDF)

## References

[1] Hatje V., Sarin M., Sander S. G., Omanović D., Ramachandran P., Völker C., Barra R. O., Tagliabue A. (2022). Emergent Interactive
Effects of Climate Change and Contaminants in Coastal and Ocean Ecosystems. Front. Mar. Sci..

[2] Moe S. J., De Schamphelaere K., Clements W. H., Sorensen M. T., Van den
Brink P. J., Liess M. (2013). Combined and Interactive Effects
of Global Climate Change and Toxicants on Populations and Communities. Environ. Toxicol. Chem..

[3] Brinkmann M., Hudjetz S., Kammann U., Hennig M., Kuckelkorn J., Chinoraks M., Cofalla C., Wiseman S., Giesy J. P., Schäffer A., Hecker M., Wölz J., Schüttrumpf H., Hollert H. (2013). How Flood Events Affect Rainbow Trout:
Evidence of a Biomarker Cascade in Rainbow Trout after Exposure to
PAH Contaminated Sediment Suspensions. Aquat.
Toxicol..

[4] Hooper M. J., Ankley G. T., Cristol D. A., Maryoung L. A., Noyes P. D., Pinkerton K. E. (2013). Interactions between Chemical and
Climate Stressors:
A Role for Mechanistic Toxicology in Assessing Climate Change Risks. Environ. Toxicol. Chem..

[5] Noyes P. D., McElwee M. K., Miller H. D., Clark B. W., Van Tiem L. A., Walcott K. C., Erwin K. N., Levin E. D. (2009). The Toxicology of
Climate Change: Environmental Contaminants in a Warming World. Environ. Int..

[6] Heugens E. H. W., Jager T., Creyghton R., Kraak M. H. S., Hendriks A. J., Van Straalen N. M., Admiraal W. (2003). Temperature-Dependent Effects of
Cadmium on *Daphnia Magna*: Accumulation
versus Sensitivity. Environ. Sci. Technol..

[7] Mangold-Döring A., Huang A., van Nes E. H., Focks A., van den
Brink P. J. (2022). Explicit Consideration of Temperature Improves Predictions
of Toxicokinetic–Toxicodynamic Models for Flupyradifurone and
Imidacloprid in Gammarus Pulex. Environ. Sci.
Technol..

[8] Raths J., Švara V., Lauper B., Fu Q., Hollender J. (2023). Speed It up:
How Temperature Drives Toxicokinetics of Organic Contaminants in Freshwater
Amphipods. Global Change Biol..

[9] Wang Z., Lui G. C. S., Burton G. A., Leung K. M. Y. (2019). Thermal Extremes
Can Intensify Chemical Toxicity to Freshwater Organisms and Hence
Exacerbate Their Impact to the Biological Community. Chemosphere.

[10] Trudgill D. L., Honek A., Li D., Van Straalen N. M. (2005). Thermal
Time – Concepts and Utility. Ann. Appl.
Biol..

[11] Cedergreen N., Nørhave N. J., Nielsen K., Johansson H. K. L., Marcussen H., Svendsen C., Spurgeon D. J. (2013). Low Temperatures
Enhance the Toxicity of Copper and Cadmium to Enchytraeus Crypticus
through Different Mechanisms. Environ. Toxicol.
Chem..

[12] Honkanen J. O., Rees C. B., Kukkonen J. V. K., Hodson P. V. (2020). Temperature Determines
the Rate at Which Retene Affects Trout Embryos, Not the Concentration
That Is Toxic. Aquat. Toxicol..

[13] Di
Toro D. M., McGrath J. A., Hansen D. J. (2000). Technical Basis
for Narcotic Chemicals and Polycyclic Aromatic Hydrocarbon Criteria.
I. Water and Tissue. Environ. Toxicol. Chem..

[14] McGrath J. A., Parkerton T. F., Hellweger F. L., Di Toro D. M. (2005). Validation of the
Narcosis Target Lipid Model for Petroleum Products: Gasoline as a
Case Study. Environ. Toxicol. Chem..

[15] Mackay D., Arnot J. A., Wania F., Bailey R. E. (2011). Chemical Activity
as an Integrating Concept in Environmental Assessment and Management
of Contaminants. Integr. Environ. Assess. Manage..

[16] Schwarzenbach, R. P. ; Gschwend, P. M. ; Imboden, D. M. Environmental Organic Chemistry, 2nd ed.; Wiley, 2005.

[17] De
Coen W. M., Janssen C. R. (1997). The Use of Biomarkers in *Daphnia Magna* Toxicity Testing. IV. Cellular Energy
Allocation: A New Methodology to Assess the Energy Budget of Toxicant-Stressed *Daphnia* Populations. J. Aquat. Ecosyst.
Stress Recovery.

[18] Ek C., Karlson A. M. L., Hansson S., Garbaras A., Gorokhova E. (2015). Stable Isotope
Composition in *Daphnia* Is Modulated by Growth, Temperature,
and Toxic Exposure: Implications for Trophic Magnification Factor
Assessment. Environ. Sci. Technol..

[19] Khattak H. K., Prater C., Wagner N. D., Frost P. C. (2018). The Threshold Elemental
Ratio of Carbon and Phosphorus of *Daphnia Magna* and Its Connection to Animal Growth. Sci.
Rep..

[20] Ek C., Garbaras A., Yu Z., Oskarsson H., Wiklund A.-K. E., Kumblad L., Gorokhova E. (2019). Increase in
Stable Isotope Ratios Driven by Metabolic Alterations in Amphipods
Exposed to the Beta-Blocker Propranolol. PLoS
One.

[21] Steigerwald S., Saladin Y., Alurralde G., Abel S., Sobek A., Eriksson Wiklund A.-K., Gorokhova E. (2025). Enhanced Tolerance to Narcosis in
Starved *Daphnia Magna* Neonates. Environ. Toxicol. Chem..

[22] Gorokhova E. (2018). Individual
Growth as a Non-Dietary Determinant of the Isotopic Niche Metrics. Methods Ecol. Evol..

[23] Zanden M. J. V., Clayton M. K., Moody E. K., Solomon C. T., Weidel B. C. (2015). Stable
Isotope Turnover and Half-Life in Animal Tissues: A Literature Synthesis. PLoS One.

[24] del
Rio C. M., Wolf N., Carleton S. A., Gannes L. Z. (2009). Isotopic
Ecology Ten Years after a Call for More Laboratory Experiments. Biol. Rev..

[25] Checkley D. M., Entzeroth L. C. (1985). Elemental
and Isotopic Fractionation
of Carbon and Nitrogen by Marine, Planktonic Copepods and Implications
to the Marine Nitrogen Cycle. J. Plankton Res..

[26] DeNiro M. J., Epstein S. (1978). Influence of Diet on the Distribution of Carbon Isotopes
in Animals. Geochim. Cosmochim. Acta.

[27] Masclaux H., Richoux N. B. (2017). Effects of Temperature and Food Quality on Isotopic
Turnover and Discrimination in a Cladoceran. Aquat. Ecol..

[28] Elendt B. P., Bias W. R. (1990). Trace Nutrient Deficiency in *Daphnia
Magna* Cultured in Standard Medium for Toxicity Testing.
Effects of the Deficiency on Life History Parameters. Aquat. Toxicol..

[29] OECD. Test No. 202: Daphnia Sp. Acute Immobilisation Test. 2004, 10.1787/9789264069947-en.

[30] Gobas F. A. P. C., Mayer P., Parkerton T. F., Burgess R. M., van de
Meent D., Gouin T. (2018). A Chemical Activity Approach to Exposure
and Risk Assessment of Chemicals. Environ. Toxicol.
Chem..

[31] Wauchope R.
D., Getzen F. W. (1972). Temperature
Dependence of Solubilities in Water and
Heats of Fusion of Solid Aromatic Hydrocarbons. J. Chem. Eng. Data.

[32] Çelik G., Healy S. A., Stolte S., Mayer P., Markiewicz M. (2025). *Daphnia Magna* as an Alternative Model for (Simultaneous)
Bioaccumulation and Chronic Toxicity Assessment—Controlled
Exposure Study Indicates High Hazard of Heterocyclic PAHs. Environ. Sci. Technol..

[33] Hoekstra J. A. (1987). Acute Bioassays
with Control Mortality. Water, Air, Soil Pollut..

[34] Post D. M., Layman C. A., Arrington D. A., Takimoto G., Quattrochi J., Montaña C. G. (2007). Getting to the Fat of the Matter: Models, Methods and
Assumptions for Dealing with Lipids in Stable Isotope Analyses. Oecologia.

[35] Anderson M. J. (2001). A New Method
for Non-Parametric Multivariate Analysis of Variance: Non-Parametric
MANOVA for Ecology. Austral Ecol..

[36] Honda M., Suzuki N. (2020). Toxicities of Polycyclic
Aromatic Hydrocarbons for
Aquatic Animals. Int. J. Environ. Res. Public
Health.

[37] Incardona J. P. (2017). Molecular
Mechanisms of Crude Oil Developmental Toxicity in Fish. Arch. Environ. Contam. Toxicol..

[38] Jiang X., Kopp-Schneider A. (2014). Summarizing
EC50 Estimates from Multiple Dose-Response
Experiments: A Comparison of a Meta-Analysis Strategy to a Mixed-Effects
Model Approach. Biom. J..

[39] Ialongo C. (2017). The Logic
of Equivalence Testing and Its Use in Laboratory Medicine. Biochem. Med..

[40] Chow S.-C. (2014). Bioavailability
and Bioequivalence in Drug Development. WIREs
Comput. Stat..

[41] van
den Berg R. A., Hoefsloot H. C., Westerhuis J. A., Smilde A. K., van der Werf M. J. (2006). Centering, Scaling, and Transformations:
Improving the Biological Information Content of Metabolomics Data. BMC Genomics.

[42] Wood, S. Mgcv: Mixed GAM Computation Vehicle with Automatic Smoothness Estimation 2000; Vol. 1, p 9 10.32614/CRAN.package.mgcv.

[43] Muijs B., Jonker M. T. O. (2009). Temperature-Dependent Bioaccumulation
of Polycyclic
Aromatic Hydrocarbons. Environ. Sci. Technol..

[44] Power M., Guiguer K. R. R. A., Barton D. R. (2003). Effects of Temperature on Isotopic
Enrichment in *Daphnia Magna*: Implications
for Aquatic Food-Web Studies. Rapid Commun.
Mass Spectrom..

[45] Camp A. A., Buchwalter D. B. (2016). Can’t
Take the Heat: Temperature-Enhanced Toxicity
in the Mayfly *Isonychia Bicolor* Exposed to the Neonicotinoid
Insecticide Imidacloprid. Aquat. Toxicol..

[46] Rombough, P. J. The Effects of Temperature on Embryonic and Larval Development. In Global Warming: Implications for Freshwater and Marine Fish; Wood, C. M. ; McDonald, D. G. , Eds.; Cambridge University Press: Cambridge, 1997; pp 177–224.

[47] Heugens E. H. W., Hendriks A. J., Dekker T., Straalen N. M. van., Admiraal W. (2001). A Review of the Effects of Multiple Stressors on Aquatic
Organisms and Analysis of Uncertainty Factors for Use in Risk Assessment. Crit. Rev. Toxicol..

[48] Valladares S., Planas M. (2020). Application of Effective Day Degrees in the Assessment
of Stable Isotope Patterns in Developing Seahorses under Different
Temperatures. Animals.

[49] Labbé K., LeBon L., King B., Vu N., Stoops E. H., Ly N., Lefebvre A. E. Y. T., Seitzer P., Krishnan S., Heo J.-M., Bennett B., Sidrauski C. (2024). Specific Activation
of the Integrated Stress Response Uncovers Regulation of Central Carbon
Metabolism and Lipid Droplet Biogenesis. Nat.
Commun..

[50] Cabral H., Fonseca V., Sousa T., Costa Leal M. (2019). Synergistic
Effects of Climate Change and Marine Pollution: An Overlooked Interaction
in Coastal and Estuarine Areas. Int. J. Environ.
Res. Public Health.

[51] Schäfer R. B., Jackson M., Juvigny-Khenafou N., Osakpolor S. E., Posthuma L., Schneeweiss A., Spaak J., Vinebrooke R. (2023). Chemical Mixtures
and Multiple Stressors: Same but Different?. Environ. Toxicol. Chem..

[52] Honkanen J. O., Kukkonen J. V. K. (2006). Environmental
Temperature Changes Uptake Rate and Bioconcentration
Factors of Bisphenol A in Tadpoles of Rana Temporaria. Environ. Toxicol. Chem..

