# Supplementary material for: When Heat Meets Pollutants: Integrating Degree-Days and Chemical Activity Concepts for the Assessment of Temperature-Driven Toxicity
Source: Environ Sci Technol. 2026 Feb 20;60(9):6916–26. doi: 10.1021/acs.est.5c10332 (PMC12980837; doi:10.1021/acs.est.5c10332)
Supplement: Supplementary file 2 [file es5c10332_si_002.pdf]

Supporting Information

When heat meets pollutants: integrating degree-days and chemical activity concepts for assessment of temperature-driven toxicity

Elena Gorokhova[1]*, Gastón Alurralde[1,2], Sophie Steigerwald[1], Sebastian Abel[1], Yves Saladin[1], Anna Sobek[1], Ann-Kristin Eriksson-Wiklund[1], Andrius Garbaras[3]

[1]Department of Environmental Science, Stockholm University, Sv. Arrhenius v. 8, 106 91 Stockholm, Sweden

[2]Baltic Marine Environment Protection Commission, HELCOM, 00160 Helsinki, Finland

[3]Mass Spectrometry Laboratory, center for Physical Science and technology, Saulėtekio al. 3, Vilnius 10257, Lithuania

*elena.gorokhova@aces.su.se

Supporting Information Summary
Number of pages: 13
Number of texts: 4 (Texts S1–S4)
Number of tables: 8 (Tables S1–S8)

Contents

Text S1: PAH dosing strategy and analytical verification 3
PAH stocks 3
Passive dosing system 3
PAH concentration measurements 3
Text S2: Chemical activity as a unified dose metric 4
Dose Metrics 4
Calculation of chemical activity and temperature adjustment 4
Text S3: Degree-days calculation 5
Text S4. Rationale for statistical testing of dose-response results 5
Model framework 5
Assessing treatment differences (question 1) 6
Assessing equivalence (question 2) 6

Integration of the two approaches...7

Table S1. PAH concentrations (C; mg/L) and activities (*a*) in each treatment and experimental run conducted under the specified conditions of temperature and exposure duration (see Table 1). 8

Table S2. Solubility values at 20°C and 25°C. 10

Table S3. Sensitivity analysis showing how La50 estimates vary across individual and pooled runs within a treatment. 11

Table S4. Estimated La_{50} values with 95 % confidence intervals from a joint four-parameter logistic model with shared asymptotes and Hill slope across treatments...12

Table S5. Bootstrap-based uncertainty estimates for treatment differences in La50. 12

Table S6. Significance and equivalence testing of La50 ratios between treatments. 13

Table S7. Interpretation of temperature × contaminant interactions based on the integration of D° normalization and chemical activity and insights from metabolic indicators. 14

Table S8. Integration of degree-day normalization, chemical activity, and physiological biomarkers into toxicokinetic–toxicodynamic (TK–TD) modeling frameworks. 15

References...15

Text S1: PAH dosing strategy and analytical verification

PAH stocks

Stock solutions (max. solubility) of all four PAHs (ACE, FLU, PHE, FluO) were prepared by dissolving the PAH crystals in methanol. From these single stocks, a mixture stock solution (in methanol) at chemical activity 0.15 at room temperature was prepared, with each compound contributing 25% of the total. This mixture stock was diluted with methanol to achieve the target chemical activities (0.01-0.1).

Passive dosing system

For the passive dosing system, 1g of silicone (PDMS, AlteSil, UK) per 20 mL of exposure medium was used. The silicone was cut into 8 equal pieces to fit inside the vials. The silicone rods were cleaned in three consecutive steps: first, they were washed in ethyl acetate for 24 h, then in ethanol for 24 h, and finally ethanol was removed by evaporation for 24 h at room temperature, followed by drying for 4 h at 120°C.

After cleaning, the rods were soaked in the PAH-methanol solutions for 72 h on a benchtop shaker. Once soaked, they were rinsed with Milli-Q water and then used to equilibrate the exposure medium (M7) for another 72 h, also on a benchtop shaker at the relevant exposure temperatures (20°C or 25°C). After this equilibration period, the vials containing the rods and M7 were ready to use in the exposure experiments.

PAH concentration measurements

After exposure, the M7 medium was discarded and the silicone rods were rinsed with Milli-Q water to remove any biological debris. The rods were then re-equilibrated in fresh Milli-Q for 72 h at the relevant exposure temperature on a benchtop shaker. Following equilibration, 500 µL of the Milli-Q extract was mixed in a 1:1 ratio with acetonitrile (500 µL) and PAH fluorescence was measured using high-performance liquid chromatography with photodiode array detection (HPLC-PDA, Shimadzu i-Series LC 2040C 3D, Shimadzu, Sweden). The PDA wavelengths used for detection were 225 (ACE), 261 (FLU), 249 (PHE), and 234 nm (FluO).

A 50 µl sample (1:1 with acetonitrile) was injected into the system, and PAHs were separated using a HALO 90 Å PAH column (particle size 2.7 µm, 2.1 x 50 mm, HALO, USA) at 30°C, operated at a flow rate of 0.5 ml/min. The mobile phases consisted of Milli-Q water (A) and acetonitrile (B), with the following gradient program: 50% B from 0 – 0.31 min, a linear increase to 70% B from 0.31 – 5.0 min, a further increase to 99% B from 5.0 – 5.2 min, maintaining 99% B until 6.5 min, followed by a linear return to 50% B within 0.1 min, and a final 1.2 min hold at 50% B. The total runtime was 8 minutes.

PAH quantification was performed using an external calibration curve prepared using the four PAHs at eight concentration points ranging 1-1000 µg/l. The determined PAH concentrations (Table S1) were used to calculate chemical activity, which was temperature-adjusted to the exposure settings (20°C or 25°C) as described in Text S2.

Text S2: Chemical activity as a unified dose metric

Dose Metrics

For non-polar HOC mixtures, chemical activity integrates all individual compounds into a single measure, reflecting their cumulative bioavailability and toxicity potential. Therefore, using chemical activity as a dose metric, we standardized exposure across different compounds, thereby removing the confounding effects of individual solubilities and concentrations, and providing a mechanistically relevant measure of mixture bioavailability and toxicity potential. This integration enables the assessment of the additive effects of multiple HOCs collectively.

Calculation of chemical activity and temperature adjustment

The maximum chemical activity (a_{max}) of each compound was calculated using Eqs. S1 and S2. This represents the chemical activity when a substance is at its solubility limit (S) at a given temperature (T). According to Gobas and co-workers (Gobas et al., 2018), a_{max} can be approximated using the melting point (T_m) of the compound.

To determine the subcooled liquid solubility (S_L) of a compound under saturated conditions, Eq. S1 was applied by substituting a_{max} for a and S for the concentration C:

$$a = {}^{C}/_{S_L} \qquad \text{(Eq. S1)}$$

Additionally, Eq. S2 relates a_{max} to the melting point and temperature of the compound:

$$a_{max} = \exp\left[-6.8 \times \left(\frac{T_m}{T} - 1\right)\right] = S/S_L \qquad \text{(Eq. S2)}$$

It is important to note that all physicochemical parameters involved in chemical activity calculations (Eqs. S1 and S2) are temperature-dependent. However, PAH solubility data are often available only at the standard temperature of 25°C.

To account for temperature-dependent solubility, we used data from Wauchope and Getzen (Wauchope and Getzen, 1972) to estimate solubility values for the experimental temperatures (25°C and 20°C; Table S1). For ACE, FLU and PHE, we applied a log-linear regression model, where solubility follows an exponential relationship with temperature (up to 75°C). The regression slope was derived for each compound using least-squares regression and interpolation of solubilities at the desired temperatures. Since FluO was not included in the dataset of Wauchope and Getzen (1972), we used published solubility values: 0.20 (https://gost.tpsgc-pwgsc.gc.ca/Contfs.aspx?ID=39&lang=eng) and 0.26 (https://www.atamanchemicals.com/fluoranthene_u26714/) for 20 °C and 25 °C, respectively (Table S2). The temperature-adjusted solubility values were used to calculate chemical activities in the exposure medium by applying Eq. S1, where the measured PAH concentrations (Table S1)

in the exposure medium were used in concert with the corresponding temperature-specific S_L values.

Text S3: Degree-days calculation

Degree-days (D°) were computed as cumulative °C·days above the baseline temperature, following the general approach of (Trudgill et al., 2005):

$$D° = \Sigma \max(0, T - T_{base}) \times \Delta t \qquad \text{(Eq. S3)},$$

where T is the exposure temperature (°C), T_{base} = 0 °C for *Daphnia magna* (Sun et al., 2023), and Δt is the duration in days.

In this study, degree-day values were calculated as:

20 °C for 72 h: 20 × (72/24) = 60 D°

25 °C for 57.6 h: 25 × (57.6/24) = 60 D°

At 25 °C, the 60 D° exposure equalled 57.6 h, reported as 58 h in the text for clarity. These two treatments, therefore, represent equivalent cumulative thermal exposure.

Text S4. Rationale for statistical testing of dose-response results

The statistical analysis of the dose-response data aimed to evaluate both differences and equivalence in La50 values among treatments. This dual approach was necessary because the study addressed two complementary questions:

(1) *whether temperature and exposure regime produced statistically different La50 values*, and

(2) *whether treatments standardized by degree-days (D°) could be regarded as biologically equivalent in their toxicity response*.

Model framework

Both individually fitted models and the joint model were calculated to capture complementary aspects of the data. The individual treatment fits provide an unconstrained, descriptive view of each dose–response curve, allowing visual inspection of treatment-specific curve shapes and slopes (Figure 2A, Table 4). However, because the treatments differed in the number of observations (12 data points per D60 treatment versus 6 in D75), the joint four-parameter logistic model was used to provide a statistically balanced comparison. In this model, the lower and upper asymptotes and Hill slope were shared across treatments, allowing the less replicated D75 dataset to borrow strength from the better-resolved D60 treatments, while La50 was estimated separately for each (Table S4).

In all dose–response models, the lower and upper asymptotes were fixed at 0% and 100% immobilization. When controls showed (near) 0% immobilization and high doses resulted in (near) 100% immobilization (Figure 2A), fixing the bounds at these values is justified and helps

obtain robust estimates of the slope and La50 (Motulsky and Christopoulos, 2004). Potential bias from this constraint was evaluated by fitting alternative models in which both asymptotes were estimated freely and comparing La50 estimates and overall fit. In these unconstrained fits, Bottom and Top were weakly informed by the data, deviating slightly from the expected range (Bottom –0.5% to 9%; Top 101–125%) and associated with wide confidence intervals, indicating limited support for estimating these parameters independently. Importantly, La50 estimates were very similar between constrained and unconstrained models: for T20_H72_D60, 0.065 (free) vs. 0.064 (fixed); for T25_H72_D75, 0.040 vs. 0.033; and for T25_H58_D60, 0.064 vs. 0.062. These differences are small relative to the dose range and indicate that fixing the asymptotes did not substantially bias treatment-specific La50 estimates, while providing more stable parameterization.

The La50 estimates obtained from the individually fitted models (Table 4) were highly consistent with those derived from the joint model (Table S4), with only minor differences in variability (Figure 2B), indicating that the shared-parameter structure captured the main treatment effects without distorting the independently observed dose-response patterns. This approach stabilizes parameter estimation and ensures that differences in La50 reflect biological rather than sampling variation. Together, the two modeling approaches capture both the empirical variability among individual fits and the robust, model-based inference across treatments with unequal sample size.

Assessing treatment differences (question 1)

The difference testing quantifies whether treatments are statistically distinguishable in terms of their La50. Two complementary methods were applied:

(a) **Bootstrap robustness check** (Table S5): A parametric bootstrap approach was implemented to evaluate the stability of the La50 variability (Figure 2B). From 5,000 resampled parameter vectors drawn from the model's asymptotic covariance, La50 values and their pairwise absolute differences were recalculated, yielding empirical 95 % percentile intervals (2.5-97.5 %). These intervals account for uncertainty in the original scale and confirm that the observed differences are robust to sampling variation and model assumptions.

(a) **Analytical inference** (Table S6): Pairwise Wald tests on log-transformed La50 values were used to evaluate the null hypothesis of no difference in La50 between treatments. The La50 ratios, their 95 % confidence intervals, Z statistics, and two-sided p-values provide formal evidence of significant and non-significant differences.

Assessing equivalence (question 2)

While difference testing in Table S6 identifies a statistically significant separation between treatments, equivalence testing evaluates whether two treatments are sufficiently similar that any difference is biologically negligible. For this, the two one-sided tests (TOST) procedure was applied to the La50 ratios (greyed area in Table S6). In TOST, equivalence is assessed at α = 0.05; accordingly, 90 % confidence intervals were used, as they correspond to the joint acceptance region of the two one-sided tests (Ialongo, 2017). A 90 % confidence interval fully contained within the pre-defined equivalence bounds of 0.80-1.25 (± 20 %) indicates that treatments are statistically equivalent in La50. The ±20 % margin was selected because it reflects

typical experimental and biological variability observed in ecotoxicological bioassays and aligns with bioequivalence standards used in pharmacological testing (Chow, 2014). This analysis directly addresses the study hypotheses that degree-day normalization compensates for temperature-related metabolic differences.

Integration of the two approaches

Together, Tables S5 and S6 provide a coherent analytical framework, where Table S5 reports bootstrap-derived confidence intervals for absolute La50 differences, visualizing uncertainty in a model-independent way. Table S6 presents the formal hypothesis testing (Wald statistic and TOST) on the ratio scale, providing significance and equivalence decisions. This two-layer strategy strengthens confidence in the results by combining empirical uncertainty quantification (Table S5) with model-based inference (Table S6). Both approaches converged on the same conclusions:

(1) La50 values for T25_H72_D675 differ significantly from both D°-normalized treatments, and

(2) T20_H72_D60 and T25_H58_D60 are statistically equivalent within ± 20 %, confirming that degree-day normalization effectively compensates for temperature-related variation in toxicity.

Table S1. PAH concentrations (C; mg/L) and activities (*a*) in each treatment and experimental run conducted under the specified conditions of temperature and exposure duration (see Table 1).

Treatment code	Run	Total activity	ACE		FLU		PHE		FluO	
			C	*a*	C	*a*	C	*a*	C	*a*
T20_H72_D60	1	0.000	0.000	0.000	0.000	0.000	0.000	0.000	0.000	0.000
		0.010	0.039	0.002	0.039	0.003	0.017	0.003	0.003	0.002
		0.038	0.141	0.008	0.140	0.011	0.063	0.011	0.013	0.008
		0.048	0.183	0.011	0.177	0.014	0.078	0.014	0.016	0.010
		0.071	0.256	0.015	0.257	0.020	0.117	0.020	0.025	0.015
		0.110	0.402	0.024	0.399	0.031	0.180	0.031	0.039	0.023
	2	0.000	0.000	0.000	0.000	0.000	0.000	0.000	0.000	0.000
		0.006	0.024	0.001	0.022	0.002	0.011	0.002	0.002	0.001
		0.037	0.136	0.008	0.122	0.010	0.065	0.011	0.013	0.008
		0.043	0.167	0.010	0.145	0.011	0.074	0.013	0.015	0.009
		0.060	0.234	0.014	0.204	0.016	0.105	0.018	0.020	0.012
		0.080	0.314	0.019	0.271	0.021	0.139	0.024	0.027	0.016
T25_H72_D75	3	0.000	0.000	0.000	0.000	0.000	0.000	0.000	0.000	0.000
		0.004	0.017	0.001	0.025	0.002	0.011	0.002	NA	NA
		0.024	0.089	0.005	0.105	0.007	0.052	0.008	0.008	0.004
		0.034	0.123	0.007	0.141	0.009	0.070	0.011	0.012	0.006
		0.052	0.183	0.010	0.209	0.014	0.106	0.017	0.020	0.011
		0.081	0.283	0.015	0.322	0.021	0.165	0.027	0.034	0.018
T25_H58_D60	4	0.000	0.000	0.000	0.000	0.000	0.000	0.000	0.000	0.000
		0.008	0.031	0.002	0.032	0.002	0.015	0.002	0.003	0.002
		0.025	0.098	0.005	0.106	0.007	0.045	0.007	0.010	0.005
		0.038	0.155	0.008	0.151	0.010	0.070	0.011	0.015	0.008
		0.065	0.258	0.014	0.253	0.017	0.120	0.019	0.027	0.014
		0.084	0.343	0.019	0.332	0.022	0.155	0.025	0.035	0.019
	5	0.000	0.000	0.000	0.000	0.000	0.000	0.000	0.000	0.000
		0.033	0.138	0.008	0.120	0.008	0.063	0.010	0.013	0.007

		0.047	0.192	0.010	0.172	0.011	0.094	0.015	0.019	0.010
		0.065	0.267	0.015	0.237	0.016	0.128	0.021	0.026	0.014
		0.103	0.426	0.023	0.375	0.025	0.204	0.033	0.041	0.022
		0.093	0.422	0.023	0.349	0.023	0.157	0.025	0.041	0.022

Table S2. Solubility values at 20°C and 25°C.

Solubilities were estimated using the semi-empirical approach of Wauchope and Getzen (Wauchope and Getzen, 1972) and a log-linear regression between solubility and temperature. Values for FluO were adopted from other sources (see Text S2) because this congener was not considered in the study of Wauchope and Getzen.

Compound	Slope	a_{max} at 20°C	Solubility at 20°C	a_{max} at 25°C	Solubility at 25°C
ACE	0.048	0.17	2.88	0.20	3.67
FLU	0.051	0.11	1.41	0.12	1.82
PHE	0.053	0.15	0.86	0.18	1.12
FluO		0.12	0.20	0.14	0.26

Table S3. Sensitivity analysis showing how La50 estimates vary across individual and pooled runs within a treatment.

The between-run variability in La_{50} values was ~15 % for T20_H72_D60 and ~4 % for T25_H58_D60, placing both at the low end of the intra-laboratory variation typically reported for acute *Daphnia* assays (≈ 8–40 % CV; (USEPA, 2002)). Similar variation among replicate trials has been observed in other chemical-activity-based studies, e.g., (Oliveira dos Anjos et al., 2023); see their Figure 2).

(A) **Runs 1 & 2, T20_H72_D60** treatment produced comparable sigmoidal responses but minor mid-range variability. The pooled fit (bold face), including all data, yielded a robust treatment-level estimate (La50 = 0.064; Table 4, Figure 2). Excluding one mid-range point (Run 1 at a = 0.0705, 25 % mortality) reduced La50 by ≈ 0.01 and produced an implausibly steep slope, confirming that retaining the point better represents experimental variability.

(B) **Runs 4 & 5, T25_H58_D60** treatment produced almost identical sigmoidal dose–response patterns. The overall fit (La50 = 0.062; 95 % CI 0.053–0.067) is reported in Table 4 and Figure 2.

Model	Data used	La50	95% CI	Hill slope	R^2	n	Notes
**A)** Runs 1 & 2, T20_H72_D60 treatment							
Run 1 (separate)	Run 1 only	0.071	0.052–0.101	6.74	0.98	5	Slightly right-shifted curve; higher mid-range mortality variability
Run 2 (separate)	Run 2 only	0.056	0.053–0.059	13.14	0.99	5	Slightly steeper curve; lower mid-range response
Pooled (no outlier)	Runs 1 + 2, Run 1 point at a = 0.0705 (the outlier) excluded	0.057	0.055–0.058	16.06	0.99	9	Unrealistically steep slope and over-narrow CIs
**Pooled (all data)**	**Runs 1 + 2, all points included**	**0.064**	**0.055–0.075**	**5.97**	**0.89**	**10**	**Treatment-level fit, all variability is included**
**B)** Runs 4 and 5, T25_H58_D60 treatment							
Run 4 (separate)	Run 4 only	0.060	0.056–0.065	11.13	0.986	5	Slightly steeper mid-range slope
Run 5 (separate)	Run 5 only	0.065	0.060–0.070	7.89	0.981	5	Near-identical curve; minor right shift
**Pooled (all data)**	**Runs 4 + 5, all points included**	**0.062**	**0.053–0.067**	**8.95**	**0.984**	**10**	**Treatment-level fit, all variability is included**

Table S4. Estimated La_{50} values with 95 % confidence intervals from a joint four-parameter logistic model with shared asymptotes and Hill slope across treatments.

This model provides directly comparable La_{50} estimates while stabilizing parameter estimation for the treatment with fewer observations.

Treatment	La50	95%-CI, low	95%-CI, high
T20_H72_D60	0.065	0.057	0.076
T25_H58_D60	0.061	0.053	0.071
T25_H72_D75	0.033	0.027	0.040

Shared parameter	Estimate (non-constrained)
Bottom	5.82
Top	98.73
Hill slope	7.01
R^2	0.90

Table S5. Bootstrap-based uncertainty estimates for treatment differences in La50.

The La50 values were estimated for each treatment using a joint four-parameter logistic model with shared asymptotes and shared Hill slope across treatments (Table S4). Uncertainty was quantified using a parametric bootstrap approach: 5000 parameter vectors were sampled from the asymptotic covariance matrix of the fitted model, transformed to La50 values, and pairwise differences were computed for each bootstrap iteration. The median differences and 95% percentile intervals (2.5-97.5%) are reported. Positive differences indicate higher La50 (lower toxicity) for the first treatment in each comparison, and the interval crossing zero for the treatments normalized to D° indicates the lack of statistical difference between the treatments.

Comparison	Median difference	2.5%	97.5%
T20_H72_D60 − T25_H58_D60	0.00443	−0.00634	0.01553
T20_H72_D60 − T25_H72_D75	0.03227	0.02277	0.04246
T25_H58_D60 − T25_H72_D75	0.02792	0.01857	0.03766

Table S6. Significance and equivalence testing of La50 ratios between treatments.

Results of pairwise Wald tests on log-transformed La50 values and equivalence testing on the La50 ratio scale, based on the joint four-parameter logistic model with shared lower and upper asymptotes and Hill slope across treatments (Table S4). For each pair of treatments, the La50 ratio (first/second) and its 95 % confidence interval (95%-CI) represent the estimated fold-difference in toxicity derived from the modeled covariance structure. The Wald Z statistic and its two-sided P-value test the null hypothesis of no difference between treatments. A significant P-value ($p < 0.05$) or a 95 % confidence interval that excludes 1 indicates a statistically significant difference.

To assess equivalence (shaded area), 90 % confidence intervals (90%-CI) of the La50 ratios were compared to pre-specified equivalence bounds of 0.80–1.25 (± 20 %). Treatments were considered equivalent when the entire 90 % confidence interval lay within these bounds (column *Equivalence, 20%;* summarizes two one-sided tests, TOST (Ialongo, 2017))).

**Comparison**	**La50 ratio**	**95%-CI, low**	**95%-CI, high**	**Wald Z**	**P value**	**90%-CI, low**	**90%-CI, high**	**Equivalence, 20%**
T20_H72_D60 / T25_H58_D60	1.072352	0.896957	1.282044	0.807227	0.4195	0.924781	1.243472	Equivalent (0.8-1.25)
T20_H72_D60 / T25_H72_D75	1.976496	1.594157	2.450534	6.541005	<0.001	1.653867	2.362062	Not equivalent
T25_H58_D60 / T25_H72_D75	1.843141	1.480525	2.294571	5.760671	<0.001	1.537055	2.210181	Not equivalent

Table S7. Interpretation of temperature × contaminant interactions based on the integration of D° normalization and chemical activity and insights from metabolic indicators.

This framework distinguishes between independent, temperature-amplified, and temperature-attenuated effects by accounting for temperature-driven physiological ageing and thermodynamically consistent contaminant exposure. When exposure durations are standardized using degree-days (D°), and dose is expressed as chemical activity, differences in toxicity outcomes can be attributed to true mechanistic interactions (e.g., altered uptake, metabolism, or elimination) rather than confounding effects of metabolic rate. Apparent additivity, synergy, or antagonism may occur under fixed-time exposure but do not represent true interactions unless they persist under D°-standardized conditions.

Interaction Type	Interpretation	Mechanistic implications	How D° + chemical activity helps
**Independent** (may appear additive under fixed-time exposure)	Toxicity (La50) does not differ between temperatures when exposures are standardized by D°.	Temperature affects metabolic rate and ageing but does not alter PAH uptake, bioavailability or toxicodynamic potency. Metabolic indicators show parallel declines across temperatures, reflecting comparable physiological stress at equivalent physiological age.	Controls for ageing and normalizes dose, showing that toxicity is independent of temperature-induced chemical behavior.
**Temperature-amplified interaction** (may appear synergistic under fixed-time exposure)	Toxicity remains higher at elevated temperature even after D° normalization (true temperature-amplified interaction).	Temperature enhances contaminant uptake, biotransformation, or target sensitivity (e.g., increased membrane permeability, enzyme activation). This results in greater metabolic disturbance at higher temperatures.	A lower La50 at higher temperature under equal D° reflects true toxicodynamic modification.
**Temperature-attenuated interaction** (may appear antagonistic under fixed-time exposure)	Toxicity is lower at elevated temperature under D°-standardized exposure.	Elevated temperature promotes detoxification, biotransformation, elimination, or reduced tissue partitioning, reducing contaminant effects.	Shows that mitigation of toxicity is due to temperature-driven physiological/kinetic processes rather than ageing differences.

Table S8. Integration of degree-day normalization, chemical activity, and physiological biomarkers into toxicokinetic–toxicodynamic (TK–TD) modeling frameworks.

Summary of how empirical data from D°-normalized exposures, chemical activity metrics, and metabolic indicators can be used to parameterize, calibrate, and validate mechanistic models such as physiologically based toxicokinetic (PBTK) and DEB models.

**Use of empirical data**	**Application in TK-TD models**
Physiological time calibration	Use D° as the time axis in TD functions to align biological processes across temperatures, consistent with observed physiological development.
Temperature-independent effect thresholds	Use chemical activity-based La50 values to define internal effect thresholds valid across temperature regimes.
Parameterization and model fitting	Fit TD models to match observed changes in metabolic indicators (e.g., $\delta^{13}C$, C:N ratio, and protein content), capturing internal damage dynamics and energy use.
Cross-validation across temperature regimes	Compare model outputs to toxicity patterns from fixed-time vs. D°-normalized assays to assess model accuracy across temperature regimes.

References

Chow, S.-C., 2014. Bioavailability and bioequivalence in drug development. WIREs Comput. Stat. 6, 304–312. https://doi.org/10.1002/wics.1310

Gobas, F.A.P.C., Mayer, P., Parkerton, T.F., Burgess, R.M., Meent, D. van de, Gouin, T., 2018. A chemical activity approach to exposure and risk assessment of chemicals. Environ. Toxicol. Chem. 37, 1235–1251. https://doi.org/10.1002/etc.4091

Ialongo, C., 2017. The logic of equivalence testing and its use in laboratory medicine. Biochem. Medica 27, 5–13. https://doi.org/10.11613/BM.2017.001

Motulsky, H., Christopoulos, A., 2004. Fitting models to biological data using linear and nonlinear regression. A practical guide to curve fitting., online. ed. Oxford Academic, New York, NY.

Oliveira dos Anjos, T.B., Abel, S., Lindehoff, E., Bradshaw, C., Sobek, A., 2023. Assessing the effects of a mixture of hydrophobic contaminants on the algae *Rhodomonas salina* using the chemical activity concept. Aquat. Toxicol. 265, 106742. https://doi.org/10.1016/j.aquatox.2023.106742

Sun, S.-J., Dziuba, M.K., Jaye, R.N., Duffy, M.A., 2023. Temperature modifies trait-mediated infection outcomes in a Daphnia–fungal parasite system. Philos. Trans. R. Soc. B Biol. Sci. 378, 20220009. https://doi.org/10.1098/rstb.2022.0009

Trudgill, D.L., Honek, A., Li, D., Van Straalen, N.M., 2005. Thermal time – concepts and utility. Ann. Appl. Biol. 146, 1–14. https://doi.org/10.1111/j.1744-7348.2005.04088.x

USEPA, 2002. Methods for measuring the acute toxicity of effluents and receiving waters to freshwater and marine organisms (5th ed., EPA-821-R-02-012). U.S. Environmental Protection Agency. Office of Water.

Wauchope, R.D., Getzen, F.W., 1972. Temperature dependence of solubilities in water and heats of fusion of solid aromatic hydrocarbons. J. Chem. Eng. Data 17, 38–41. https://doi.org/10.1021/je60052a020
